# Phytochemical, biological, and nutritional properties of the prickly pear, *Opuntia dillenii*: A review

**DOI:** 10.1016/j.jsps.2024.102167

**Published:** 2024-08-30

**Authors:** EL Hassania Loukili, Mohammed Merzouki, Mohamed Taibi, Amine Elbouzidi, Belkheir Hammouti, Krishna Kumar Yadav, Mohammad Khalid, Mohamed Addi, Mohammed Ramdani, Pankaj Kumar, Jeong Ryeol Choi

**Affiliations:** a Euromed University of Fes, UEMF, Morocco; b Laboratory of Applied Analytical Chemistry Materials and Environment (LC2AME), Faculty of Science, Mohamed First University, Oujda, Morocco; c Laboratory of Applied Chemistry and Environment (LCAE-ECOMP), Faculty of Sciences, University Mohamed Premier, Oujda, Morocco; d Laboratoire d’Amélioration des Productions Agricoles, Biotechnologie et Environnement (LAPABE), Faculté des Sciences, Université Mohammed Premier, Oujda 60000, Morocco; e Department of Environmental Science, Parul Institute of Applied Sciences, Parul University, Vadodara, Gujarat 391760, India; f Environmental and Atmospheric Sciences Research Group, Scientific Research Center, Al-Ayen University, Thi-Qar, Nasiriyah 64001, Iraq; g Department of Pharmaceutics, College of Pharmacy, King Khalid University, Asir-Abha 61421, Saudi Arabia; h School of Electronic Engineering, Kyonggi University, Yeongtong-gu, Suwon, Gyeonggi-do 16227, Republic of Korea

**Keywords:** Antioxidant and biological properties, *Opuntia*, Phytochemicals, Prickly pear

## Abstract

Opuntia (Cactaceae) species are native to arid and semi-arid regions of Mexico and the southern United States and grow in various climatic zones. *Opuntia dillenii* is a cactus fruit with many beneficial properties, and it is used as a medicinal plant in various countries. This review paper provides updated information on the phytochemical and pharmacological aspects of *O. dillenii*. The fruit contains valuable compounds such as flavonoids, phenolics, ascorbic acid, betanin, and essential elements, which have been isolated and identified. The fruit also exhibits diverse pharmacological activities, such as antioxidant, anti-inflammatory, anti-tumor, neuroprotective, hepatoprotective, hypotensive, anti-diabetic, antifungal, and anticancer effects. Moreover, molecular docking and ADMET predictions were performed to evaluate the antibacterial potential of the fruit against *Escherichia coli* protein. This paper suggests that *O. dillenii* has significant potential as a complementary therapy for various pathological conditions.

## 1 Introduction

Within the taxonomic classification of the Cactaceae family (Arba, 2009, Besné-Eseverri et al., 2023), the Opuntia species, commonly known as prickly pear, originate from the arid and semi-arid regions of Mexico and the southern United States (Abdallah, 2008, Ahmed et al., 2005, Loukili et al., 2022). During the 16th century, maritime traders facilitated the spread of Opuntia to Europe and North Africa (Bouzoubaâ et al., 2016, Kadda et al., 2022). Among its various species, *Opuntia ficus-indica* and *Opuntia dillenii* are particularly notable, extensively utilized across numerous countries for both agricultural and medicinal purposes. Of these, *Opuntia dillenii* stands out due to its significant benefits for humans, animals, and the environment, acting as a source of chemically com-plex and pharmacologically important compounds.

The genus *Opuntia* has garnered extensive global research interest due to its potential in the prevention and management of chronic diseases. In Morocco, current initiatives aim to enhance cactus cultivation and incorporate it into daily dietary practices. This necessitates a thorough analysis of the chemical composition of the edible parts of *Opuntia*, including the pulp, skin, and seeds. Additionally, *Opuntia* serves as a crucial fodder resource in arid and semi-arid regions. *Opuntia*-derived feed is a rich source of bioactive compounds, such as betalains, polyphenols, carotenoids, vitamin C, and minerals, known for their preventive properties against various diseases. These compounds exhibit significant physiological effects, including antioxidative, anticancer, and neuroprotective activities, effectively protecting cells from the harmful effects of free radicals through their inherent redox properties (Rahimi et al., 2019, Simopoulos, 2002).

Numerous investigations substantiate *O. dillenii* therapeutic efficacy in addressing a spectrum of disorders (Batool et al., 2016), coupled with its potential to confer neuroprotection against afflictions such as Alzheimer's, Parkinson's, and cerebrovascular pathologies (Saleem et al., 2005). The aqueous extract derived from *Opuntia* fruit assumes prominence as a copious repository of dietary fiber, vitamins (B1, B2, and C), and natural chromogens (betanin and indicaxanthin) (Attia et al., 2013, Butera et al., 2002, Cejudo-Bastante et al., 2014, Khan and Giridhar, 2015, Khan et al., 2015, Sandquist and McHale, 2011), which collectively engender the fruit's vivid crimson chromaticity. Moreover, *O. dillenii* stands as a distinguished entity esteemed for its analgesic, antihyperglycemic (Perfumi and Tacconi, 1996), anti-inflammatory (Park et al., 2001), antifungal (Sharavana Kumaar et al., 2013), anticancer (Feugang et al., 2006), hepatoprotective (Sharavana Kumaar et al., 2013), and antidiabetic attributes (Galati et al., 2005, Madrigal-Santillán et al., 2022). Conspicuously, the seeds of *O. dillenii* unveil potential marked by prodigious antioxidant activity, a consequence of their elevated content of polyphenolic compounds, flavonoids, and copious unsaturated fatty acids, thereby proffering coveted natural antioxidants for both the pharmaceutical and comesti-ble sectors (Calvi et al., 2023, Chang et al., 2008). Moreover, the derivative oil sourced from *O. dillenii* garners recognition for its pronounced abundance in fatty acids and vitamin E (Ciriminna et al., 2017), thereby signifying potential pertinence to cutaneous health maintenance as both anti-aging and anti-wrinkle agents. This lipidic derivative further underscores its potential as a prolific source of linoleic acid (omega-6) (Ali Alsaad et al., 2019), a factor implicated in the regulatory milieu of cardiovascular disorders. Cumulatively, these synergistic attributes have undeniably spurred the fervent exploration of *O. dillenii’s* fruit in scientific inquiry.

Drawing upon exhaustive analyses of its chemical composition, the fruit of *O. dillenii* showcases a notable abundance of fatty acids, prominently including polyunsaturated fatty acids, notably linoleic acid and oleic acid, renowned for their pronounced potential in ameliorating the susceptibility to cardiovascular, inflammatory, and autoimmune maladies (Simopoulos, 2002, Wang et al., 2022). Notably, *O. dillenii’s* distinctive hallmark is discernible in its opulent reservoir of unsaponifiable compounds, with β-sitosterol prominently featured as a pivotal constituent with a well-documented presence in medicinal botanicals (Sendker et al., 2020). Furthermore, the inclusion of γ-tocopherol, a potent antioxidant micronutrient, serves to underscore its pivotal role in counteracting the deleterious impact of free radicals stemming from routine cellular metabolic processes and diverse stressors, thereby furnishing a substantive contribution to overall health and well-being (Ban et al., 2024, Milenkovic et al., 2012). The seeds of *Opuntia dillenii* are notable for their substantial content of high-quality edible oils, offering a profusion of unsaturated fatty acids and polyphenols. These compounds embody valuable natural antioxidants with significant utility for the pharmaceutical sector. In addition to theoretical molecular docking and ADMET projections, a rigorous investigation of the antibacterial properties and stability of complexes within the active site of the *Escherichia coli* protein is imperative for a comprehensive evaluation.

This study presents an overview of the phytochemical and pharmacological properties of *O. dillenii*. We highlight the versatility and potential of *O. dillenii* as a natural source of bioactive substances that can be used for various purposes, such as food, medicine, and cosmetics. We also identify current challenges and opportunities for research and development on this plant.

## 2 Taxonomic classification

*Opuntia dillenii*, commonly known as Erect Prickly Pear or Dillen's Prickly Pear, is a cactus species native to the Americas. This succulent plant, recognized for its spiny pads and edible fruits, is taxonomically classified in Table 1(Böhm, 2008).

**Table 1:** Taxonomical classification of *Opuntia dillenii.*

Kingdom	Plantae
Phylum	Tracheophyta
Class	Magnoliopsida
Order	Caryophyllales
Family	Cactaceae
Subfamily	Opuntioideae
Genus	Opuntia
Species	*Opuntia dillenii*

## 3 Chemical composition

### 3.1 Fatty acids

The compositional analysis of *O. dillenii* seed oil underscores its classification as a poly-unsaturated oil (Simopoulos, 2002). Despite geographic variations in *Opuntia* origins, such as Morocco, Algeria, Tunisia, Turkey, Germany, Mexico, and China, linoleic acid consistently emerges as the principal fatty acid within these oils, followed by oleic acid and palmitic acid (Ennouri et al., 2005, Ramadan and Mörsel, 2003, Ramírez-Moreno et al., 2017). Notably, linoleic acid exhibits higher quantities within *O. dillenii* oil than *O. ficus* indica oil. Remarkably, *O. ficus* indica seed oil from Algeria's Ain el Rahma region boasts the highest linoleic acid content among various *Opuntia* oil sources (Ghazi et al., 2013). Noteworthy exceptions include the absence of oleic acid in *O. dillenii* seed oil from Morocco and China (Liu et al., 2009). Furthermore, minute amounts of myristic acid, palmitoleic acid, stearic acid, elaidic acid, vaccenic acid, arachidic acid, linolenic acid, behenic acid, cetoleic acid, and lignoceric acid were detected in these oils (Tlili et al., 2011). Parallel investigations have consistently highlighted the richness of *Opuntia* seeds in sterols, with ß-sitosterol reigning as the predominant sterol among its counterparts (Gharby et al., 2020).

**Fig. 1:**
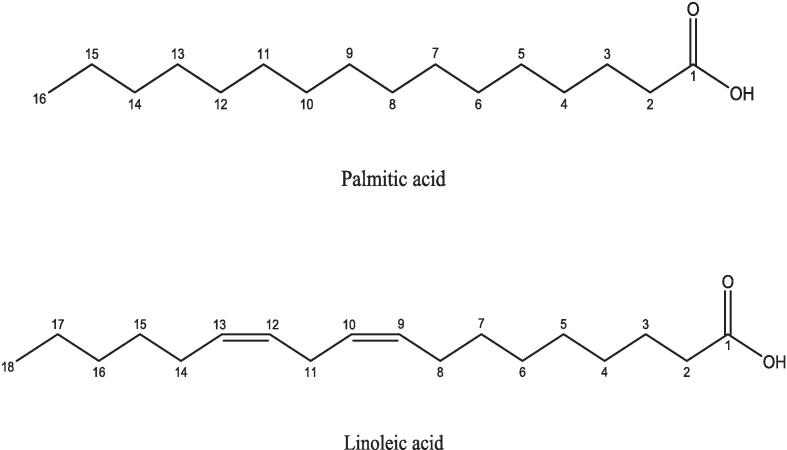
Main fatty acids, linoleic acid, and palmitic acid of Opuntia dillenii oil seeds.

In-depth analysis by Ghazi et al., (2015) has revealed that oil extracts from *O. dillenii* seeds prominently feature linoleic acid as the prevailing unsaturated fatty acid, accounting for 72.39 %, succeeded by palmitic acid, oleic acid, and stearic acid (Ghazi et al., 2015). Notably, *O. dillenii* oil's notable content of essential fatty acids and vitamin E (as depicted in Fig. 1) augments its potential utility for cutaneous applications, including anti-aging and anti-wrinkle formulations. This oil variety also serves as a potential reservoir of linoleic acid (omega-6), implicated in cardiovascular ailments' regulatory framework, while exhibiting hepatoprotective and antidiabetic attributes (Bouhrim et al., 2019, Bouhrim et al., 2018). The convergence of these diverse properties has likely positively influenced the exploration and investigation of *O. dillenii* fruits.

### 3.2 Vitamin E

**Fig. 2:**
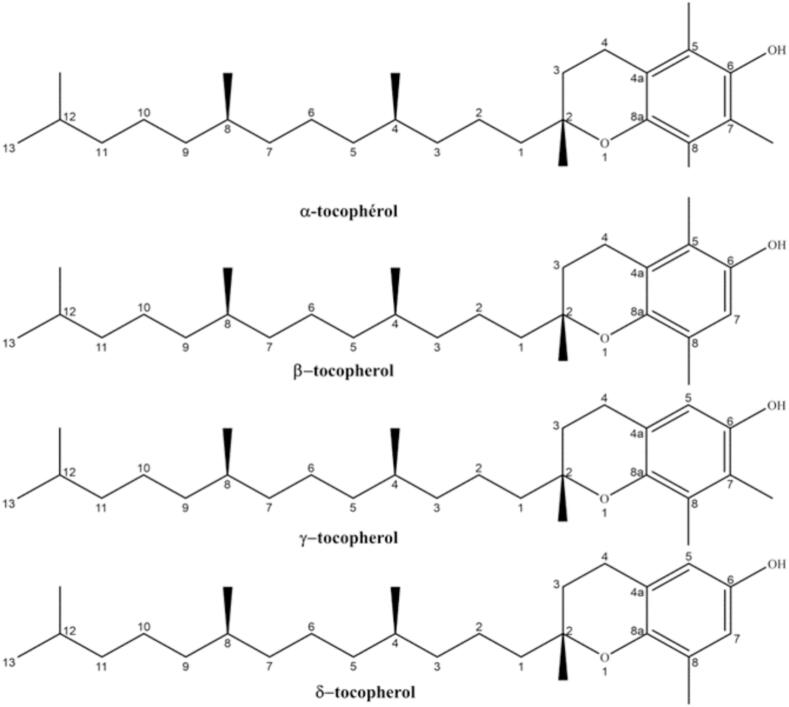
Structures of tocopherol substituents.

Vitamin E is a group of eight fat-soluble compounds that include four tocopherols and four tocotrienols. It is a crucial nutrient for maintaining various body functions. Vitamin E comprises eight different compounds, divided into two main categories: tocopherols and tocotrienols. Each category includes four forms: alpha (α), beta (β), gamma (γ), and delta (δ) (Fig. 2). Cactus seed oil is a prolific source of tocopherols, commonly known as vitamin E, with α-tocopherol and γ-tocopherol being the primary constituents. These two compounds collectively comprise ap-proximately 80 % of the total vitamin E content (El Kharrassi et al., 2020). Tocopherols are crucial for meeting the essential lipophilic requirements of the human body (Helsen et al., 2007). Tocopherols function as antioxidants by adeptly sequestering peroxide radicals within unsaturated lipids, thereby impeding the propagation of lipid peroxidation processes (Cheeseman and Slater, 1993, Traber, 2007). Their indispensable role within the physiological milieu lies in their pronounced antioxidative efficacy, which counteracts injurious chemical reactions initiated by free radicals, thus contributing to the amelioration of diverse pathological conditions.

In a study by El Kharrassi et al., (2018), *Opuntia* was found to have a remarkable total tocopherol content of 815 mg/kg, with α-tocopherol constituting 1.35 % and γ-tocopherol comprising 98 % of the total. Hensley et al., (2004) expounded upon the beneficial influence of γ-tocopherol on human nutrition and health, accentuating its potential as an effective antioxidant. These bioactive moieties function as effective scavengers of free radicals and natural antioxidants, rendering them suitable for formulation and incorporation into functional juice products (Poudel et al., 2019). Notably, *Opuntia dillenii* stands out among plant species for its abundant spectrum of tocopherol forms (α, β, γ, and δ). The vitamin E content in these seeds constitutes around 0.04 % of the oil composition. El Mannoubi et al., (2009) observed a preponderance of γ-tocopherol, accounting for 94.12 % of the overall vitamin E content, alongside a contribution of 3.42 % from δ-tocopherol. Ghazi et al., (2015) reported a vitamin E content of 0.29 % in *O. dillenii* seed oil.

### 3.3 Betanin

Food colorants are integral components of modern processed foods, and betanin is a notable natural pigment with significant utility due to its nutritional attributes (Esatbeyoglu et al., 2015). This water-soluble colorant exists in two primary forms: reddish-violet betacyanin and yellow-orange betaxanthin. Betanin is crucial for promoting human health, exhibiting a range of pharmacological activities, including antioxidant, antilipidemic, antimicrobial, antitumor, antiviral, and anticancer properties. In the context of *O. dillenii*, these attributes particularly its antioxidant, anticancer, antilipidemic, and antimicrobial properties are especially prominent (Smeriglio et al., 2019). *O. dillenii* serves as an exemplary source of edible betanin, distinguished by potent antiproliferative properties.

**Fig. 3:**
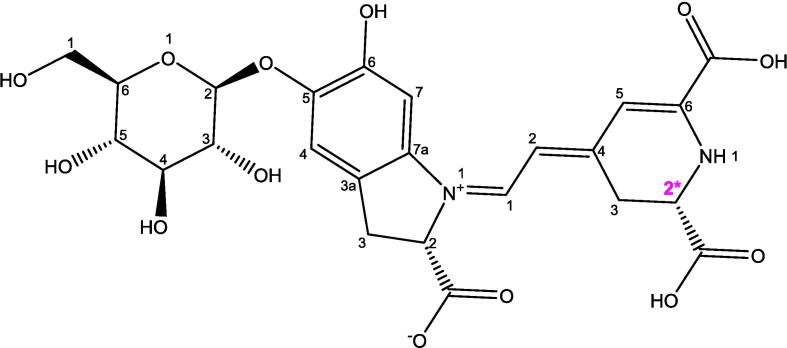
Betanin structure. *Isobetanine: C2* epimer of betanine.

Betanin and isobetanin are pigments found in plants, belonging to the class of compounds known as betalains. These pigments are responsible for the red-violet color of beets and have various applications in food coloring and potential health benefits. Isobetanin is an isomer of betanin. Isomers are compounds with the same molecular formula but different structures. The difference lies in the position of certain atoms or groups within the molecule (Fig. 3).

Extracts from prickly pear pulp, enriched with betalains, have demonstrated antibiotic efficacy in managing wounds, infections, and inflammation within the gastrointestinal and urinary tracts. Moreover, significant inhibitory effects have been observed against ovarian tumors and cervical cancer cells. The optimal pH range for betanin stability is between 5.5 and 5.8. Long-term exposure to oxygen, light in the presence of oxygen, elevated temperatures, and high-water activity can impact betanin’s stability. However, it remains remarkably stable under low humidity conditions (Smeriglio et al., 2021).

Explorations by Betancourt et al., (2017) have unveiled the chromatographic HPLC profile of *O. dillenii* fruit, revealing that the peel contains the highest concentrations of betanin and isobetanin, quantified at 15.7 and 19.2 mg/100 g of fresh sample, respectively. Intriguingly, this compound remains undetectable within the seeds. Interestingly, these compounds are undetectable in the seeds. Additionally, Betancourt et al., reported a substantial content of total betacyanins (16.63 mg/100 g of fresh fruit) and betaxanthins (7.55 mg/100 g of fresh fruit) within *O. dillenii* (Betancourt et al., 2017). Researchers have demonstrated the antioxidative potential of betanin in diverse lipid oxidation systems, further substantiating its multifaceted utility.

### 3.4 Polyphenols

Comprehensive phytochemical analyses have underscored *Opuntia’s* distinction as a polyphenol-abundant plant, with these compounds attaining central significance as fundamental constituents. Substantiated by their extensively documented antioxidant attributes (Rice-Evans et al., 1996), extracts from *O. dillenii* aptly demonstrate the capability to neutralize free radicals, consequently engendering their conversion into more stable entities. This mechanism effectively disrupts the chain reaction of radical propagation, thereby furnishing potent antioxidant prowess.

Yilmaz and Toledo's research highlights the extensive range of phenolic antioxidants, including flavonoids, tannins, coumarins, and more recently, procyanidins, which exhibit a dose-dependent ability to neutralize radicals. This positions them as promising therapeutic agents in combating pathogenic free radicals (Koubaa et al., 2017, VanderJagt et al., 2002). Notably, *O. dillenii* seeds are distinguished by their significantly high polyphenol content, followed by the skin and pulp components. Among the predominant phenolic compounds identified are quercetin, rutin, naringin, catechins, caffeic acid, gallic acid, chlorogenic acid, catechol, cinnamic acid, 3-phenylpropionic acid, psoralen, syringic acid, sinapaldehyde, 3′-O-methylcatechin, (+)-gallocatechin, bisdemethoxycurcumin, and 4′-O-methyl-(−)-epicatechin 3′-O-glucuronide, along with Viscutin (da Silva et al., 2023, Koubaa et al., 2017).

Katanić et al., (2019) have elucidated the noteworthy flavonol content within DO extracts, with variances influenced by the specific solvent employed. Aqueous extracts exhibit higher flavonol concentrations (4.5 ± 0.2 g/mg), with compounds such as quercetin and kaempferol predominantly found in skin extracts. Conversely, aqueous extracts showcase heightened quercetin content. Kaempferol emerges as the prominent flavonol within the fruit peel, with trace amounts of myricetin present across all extracts.

**Fig. 4:**
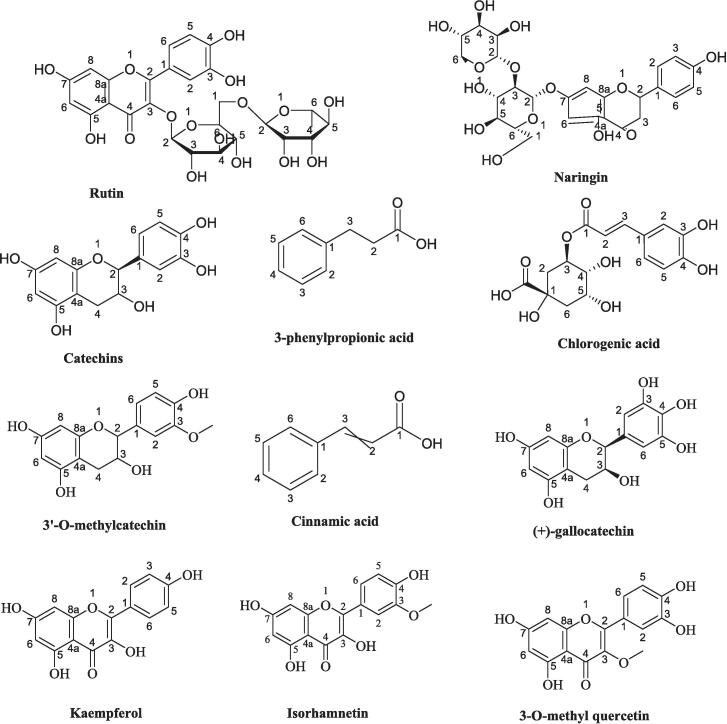
Major phenolic compounds of *Opuntia dillenii*.

Extensive investigations have corroborated the significant reservoir of polyphenols present within *Opuntia dillenii* fruit. Qiu et al., (2003) successfully isolated six compounds from *O. dillenii* stems, specifically 3-O-methyl quercetin, kaempferol, kaempferide, quercetin, isorhamnetin, and β-sitosterol, utilizing chemical evidence and spectral analysis (Fig. 4). Subsequently, a distinct study identified a novel compound, 4-ethoxy-6-hydroxymethyl-alpha-pyrone, within the ethanolic stem extract (Qiu et al., 2002). This research marked the inaugural isolation of kaempferol, kaempferide, and 3-O-methyl quercetin from *O. dillenii* stems, thus further expanding the understanding of its chemical composition. Moreover, explorations identified novel compounds, including methyl linoleate, 7-oxositosterol, 6β-hydroxystigmast-4-ene-3-one, and eucomic acid, derived from *O. dillenii* stems (Jiang et al., 2006). Furthermore, novel constituents were unveiled, such as opuntioside I, 4-ethoxy-6-hydroxymethyl-α-pyrone, and kaempferol, extracted from the stems of *O. dillenii* (Shirazinia et al., 2019).

Parallel investigations by Arba found that seeds harbor elevated levels of flavonoids and polyphenols compared to those observed in the juice and skin (Arba, 2009). These compounds, unique to the plant's secondary metabolism, contribute to its distinct phytochemical profile. Phytochemical scrutiny of mature *O. dillenii* stems by Chinedu unveiled catechin and kaempferols as the most abundant flavonoids, quantified at 44.90 µg/ml and 12.41 µg/ml, respectively. In contrast, anthocyanin and rutin had lower concentrations, measuring 0.04 µg/ml and 0.88 µg/ml, respectively (Chinedu et al., 2017).

## 4 Uses of *O. dillenii*

The prickly pear plant, characterized by its xerophytic nature, produces edible fruits and serves as livestock fodder. This makes it an ideal candidate for sustainable agricultural practices in arid and semi-arid regions, due to its inherent resilience to drought conditions and its dual contribution to human nutrition and animal husbandry. Various ecological and physiological adaptations enable its remarkable ability to thrive in water-scarce environments. Traditional usage of cactus as a medicinal resource is prevalent across various countries (Callen, 1965, Medina et al., 2007).

*Opuntia dillenii* specifically holds traditional significance in diverse regions, including Pakistan, where it addresses inflammatory conditions using preparations such as pureed leaves and milky juice, often administered at a rate of ten drops (Mahmood et al., 2011). Furthermore, it is useful in man-aging diabetes, gastric ulcers, asthma, hepatitis, intestinal spasms, ophthalmia, and whooping cough (Ying et al., 2007). Tunisian traditional medicine employs ripe *O. dillenii* fruit as an antidiabetic remedy (Medina et al., 2007). The observation of reddish urine following the consumption of these ripe fruits has led to its use in treating gonorrhea. Crushed leaves are applied as poultices for their cooling properties, effectively reducing heat and inflammation.

Moreover, the succulent leaves of the prickly pear are utilized in treating various inflamed wounds, functioning as a healing agent (Loro et al., 1999). The flowers of this plant are recognized for their efficacy in addressing bronchitis and asthma. The prickly pear also finds application in managing coughs, bronchial disorders, asthma, diabetes, gastric ulcers, and inflammation (Khan et al., 2015, Khan and Giridhar, 2015). In traditional Canadian medicine, the prickly pear fruit is an antidiabetic agent. Conversely, the prickly pear is useful across numerous regions, functioning as an anti-inflammatory, antiviral, antibacterial, antioxidant, anti-ulcer, and wound-healing agent (Brahmi et al., 2011). Within the traditional medicine of Morocco, this cactus is employed for addressing conditions including diabetes mellitus, digestive system disorders, kidney and urinary infections, allergies (Chaouch et al., 2015), type 2 diabetes, anti-aging (at-tributed to its vitamin E content), and cancer (Chavez-Santoscoy et al., 2009).

The extensive traditional medicinal applications of *O. dillenii* underscore its therapeutic potential and the need for further scientific exploration. Its ability to provide nutritional and medicinal benefits, coupled with its ecological adaptability, makes it a valuable plant for both sustainable agriculture and traditional healthcare systems.

## 5 Pharmacology of *Opuntia*

### 5.1 Antioxidant activity

*Opuntia* emerges as a significant cactus species with extensive and diverse applications in agronomy and medicine across many countries, harboring the potential to serve as a repository of a wide array of chemical compounds that hold profound biological and pharmacological significance. Prior investigations have highlighted *Opuntia's* notable antioxidative attributes (Guedes et al., 2023, Ngoc et al., 2023). A study by Brahmi et al., (2020) elucidated the exceptional antioxidative prowess inherent in *O. ficus indica* seed oil. Notably, the prickly pear seed oil displayed the highest total antioxidant activity (0.56 ± 0.01 AU), a statistically significant observation at P ≤ 0.05. This assessment employed the DPPH assay, predicated on a stable free radical (DPPH•) capacity to diminish color intensity in the presence of antioxidants. Remarkably, *O. ficus indica* seed oil extract exhibited the most robust anti-radical activity (37.0 ± 4.2 %) at 100 mg/ml concentration.

In research done for augmenting *Opuntia's* antioxidant prowess, Ramírez-Moreno et al., (2017) in Mexico rigorously explored the antioxidant activity of *O. ficus indica* seed oils obtained through distinct solvent extraction methods (hexane, ethanol, and ethyl acetate). The outcomes of this investigation unveiled substantial scavenging capabilities against the 2,2-diphenyl-1-picrylhydrazyl radical (DPPH) within these oils. Among the solvents employed, ethyl acetate-extracted oil exhibited the most elevated antioxidant activity (274 pmol TE / 20 mg of extract), followed by ethanol (247 mol TE / 20 mg of extract), with hexane registering comparatively lower values. Concurrently, a separate Mexican study utilized an ultrasound-assisted extraction technique to probe antioxidant activity, yielding results of 66.25 mg AAE / 100 g and 289 μmol TE / 100 g for 2,2′-azino-bis(3-ethylbenzothiazoline-6-sulphonic) acid (ABTS) and DPPH, respectively.

Moreover, an assessment of Moroccan *Opuntia* oil (Oujda) through a DPPH assay high-lighted its antioxidant activity surpassing that of the reference ascorbic acid (IC50 = 19.79 ± 0.023 μl / ml vs. IC50 = 16.56 ± 0.019 μg / ml). This evaluation underscored the concentration-dependent antioxidant activity of *O. ficus indica* oil, particularly sourced from its seeds (Ghazi et al., 2015). In alignment with these findings, Berraaouan et al., (2014) and Giraldo-Silva et al., (2023) reported substantial antioxidant efficacy for Moroccan *O. ficus-indica* seed oil, characterized by an IC50 value of 0.96 mg / mL. Notably, the maximal efficiency of the oil in DPPH scavenging observed at 90 min slightly trailed that of ascorbic acid [(97.12 ± 0.57) %, 1.6 mg/ml], registering at [(86.20 ± 0.13) %, 6 mg/ ml] (Ö-Zcan and Al Juhaimi, 2011).

The antioxidant potential of *O. dillenii* seed oil was rigorously evaluated using the DPPH assay and a β-carotene bleaching test. Both methods confirmed the substantial antioxidant capacity of the seed oil, nearly matching the effectiveness of standard reference compounds such as ascorbic acid and butylated hydroxytoluene (BHT). This antioxidant activity demonstrated a concentration-dependent pattern, consistent with previous findings.

In a separate investigation, the antioxidant efficacy of *O. dillenii* seed oil was specifically examined using the DPPH scavenging assay. The results revealed that *O. dillenii* seed oil (27.21 ± 0.075 μL/mL) exhibited significant antioxidant activity compared to ascorbic acid (IC50 = 16.56 ± 0.019 μg/mL), with the potency of the activity varying according to concentration (Ghazi et al., 2015). Additionally, the antioxidant potential of *O. dillenii* seed oil extracted using supercritical carbon dioxide (SC-CO_2_) or hexane was assessed, revealing substantial inhibition percentages. Using the DPPH free radical assay, the antioxidative capacity of phenolic compounds derived from *O. dillenii* seed oil was quantified. The results indicated a proportional increase in DPPH inhibition corresponding to the extract's concentration. Specifically, ascorbic acid, BHA, hexane seed oil extract, and SC-CO_2_-extracted seed oil exhibited IC50 values of 3, 5, 9, and 10 μg/ml, respectively (Ö-Zcan and Al Juhaimi, 2011).

Moreover, Katanić et al., (2019) comprehensively assessed the overall total antioxidant activity inherent in *O. dillenii* fruit extracts. Intriguingly, the highest antioxidative capacity was observed in the aqueous juice extract from the Essaouira region, Morocco. Ethanol extracts of seeds and juice from the same region displayed values approximately two-fold lower. Remarkably, ethanolic seed extracts showcased optimal DPPH scavenging potential. However, when contrasted with reference compounds like ascorbic acid, ellagic acid, and quercetin, ethanolic seed extracts from Oujda (Morocco) and Essaouira regions exhibited relatively diminished antioxidative potential against DPPH radicals. Conversely, the ethanol extracts of seeds from both Oujda and Essaouira regions excelled in scavenging ABTS radical cations, a feat mirrored by ethyl acetate and ethanolic extracts from Essaouira juice (Katanić et al., 2019).

In a study conducted by Bouhrim et al., (2020), the antioxidant activity of *O. dillenii* seed oil was further investigated. The findings unequivocally demonstrated noteworthy antioxidant activity for the cactus seed oil, characterized by an IC50 value of 0.38 ± 0.08 mg/ml. Notably, however, the IC50 value of ascorbic acid was lower (0.23 ± 0.01 μg/ml) than that of *O. dillenii* seed oil, indicative of its comparative antioxidative potency.

### 5.2 Antimicrobial activity

The antimicrobial efficacy of *O. ficus* indica seed oil has been the subject of thorough investigation across various studies. In one notable inquiry, the findings unveiled significant antibacterial activity of the ethanol-extracted oil against a range of bacterial strains, leading to appearing distinct inhibition zones. This oil exhibited remarkable antibacterial influence encompassing a broad spectrum of bacteria, including *E. coli* O58: H21 (11.4 ± 0.9 mm), *Staphylococcus aureus* (11.1 ± 1.1 mm), *Listeria monocytogenes* (11.4 ± 0.9 mm), and *Pseudomonas aeruginosa* (15.1 ± 2.0 mm) (Brahmi et al., 2011, Zhang et al., 2022).

Likewise, a separate investigation delved into the antibacterial potential of *O. ficus* indica seed oil against a gram-positive bacterium and two gram-negative bacteria. This study's results illuminated the seed oil's antibacterial prowess against *Staphylococcus aureus* and *E. coli*, while the observed effect did not extend to *Pseudomonas aeruginosa* (Ramírez-Moreno et al., 2017). Katanić et al., (2019) investigated the antimicrobial potential of *O. dillenii* fruit extracts, employing microdilution tests as the analytical approach. The study revealed a range of minimum inhibitory concentration (MIC) values, predominantly from 0.63 to 2.5 mg/ml. Notably, the aqueous seed extract of *O. dillenii* from the Nador region, Morocco, displayed the lowest MIC value of 0.31 mg/ml against *B. subtilis*. However, it is pertinent to observe that the standard antibiotic chloramphenicol exhibited significantly higher efficacy against all examined bacteria, manifesting MIC values within the range of 1.3 to 20 μg/ml. Examining the extracts of *O. dillenii* seeds from the Nador region, the study revealed the lowest antifungal MIC values, ranging from 0.16 to 2.5 mg/mL. When analyzing plant material from the Essaouira region, only ether and aqueous extracts were found to be effective against six different fungal species. Notably, the yeast *Candida albicans* exhibited significant resilience in the antifungal tests, with the *O. dillenii* extracts showing limited effectiveness (MIC > 10 mg/ml), except for the diethyl ether extract, which demonstrated positive activity with an MIC value of 10 mg/mL. Additionally, most skin extracts, except for aqueous extracts, displayed reduced efficacy at the highest concentration tested (MIC > 10 mg/ml).

### 5.3 Antidiabetic effect

Conducted within the Moroccan context, a study by Berraaouan et al., (2014) and Giraldo-Silva et al., (2023) shed light on the favorable influence of *O. ficus indica* seed oil on oral glucose tolerance. The findings unveiled a notable reduction in postprandial hyperglycemia, with decreases of 40.33 % and 16.01 % observed in healthy rats and streptozotocin-induced diabetic rats, respectively. Moreover, the oil exhibited a significant impact on diminishing intestinal glucose absorption, registering a reduction of 25.42 %. In a distinct investigation involving mice subjected to alloxan injections, applying *O. ficus indica* seed oil yielded substantial outcomes. The oil contributed to a decline in alloxan-induced mortality among the mice, concurrently mitigating the elevation of blood glucose levels and alleviating body weight loss in comparison to the untreated group. Additionally, the oil demonstrated protective effects against the alloxan-induced impairment of pancreatic islets. Oral administration of the oil notably conserved the normal state of pancreatic islets, encompassing their diameter, surface area, and the quantity of insular cells, within the alloxan-injected mice (Berraaouan et al., 2015).

An investigation in Tunisia focused on the prickly pear revealed a significant hypoglycemic effect when *O. ficus-indica* seed oil was incorporated into the rat diet. This dietary supplementation resulted in a 22 % reduction in blood glucose levels and a substantial increase in glycogen concentration in both liver and muscle tissues.

Turning to *O. dillenii* extracts, their potential in vitro antidiabetic activities were examined. A key strategy in diabetes management involves inhibiting carbohydrate-hydrolyzing enzymes (α-amylase and α-glucosidase) to delay glucose absorption and mitigate postprandial hyperglycemia. Research has aimed to identify compounds that can inhibit these enzymes for diabetes prevention and treatment. Oral administration of varying doses of *O. dillenii* seed oil to diabetic animals resulted in significant reductions in blood glucose levels, glycosuria, total cholesterol, triglycerides, liver enzymes, urea, creatinine, and uric acid. These effects were accompanied by a noticeable increase in hepatic glycogen content. The administered oil also impacted food in-take, urinary volume, and body weight loss among the treated rats, with these effects demonstrating a dose-dependent pattern (Bouhrim et al., 2019).

Furthermore, *O. dillenii* seed oil exhibited notable antidiabetic and antihyperlipidemic properties. In albino mice, this cactus seed oil was found to mitigate body weight loss, reduce elevated blood sugar levels, and decrease alloxan-induced mortality rates. Additionally, the oil improved lipid profile disturbances and countered the rise in blood glucose levels induced by a high-fat diet. These beneficial effects were associated with the oil's phenolic content and anti-oxidant activity, highlighting its potential as a valuable source of bioactive compounds with significant medicinal implications (Bouhrim et al., 2020).

### 5.4 Cytotoxic activity of *O. dillenii* extracts on cell viability

To evaluate the potential cytotoxic effects of *O. dillenii* extracts, three human cancer cell lines originating from the liver (HepG2), colon (LoVo), and breast (MCF-7) were employed. The respective IC50 values, representing the concentration required to inhibit cell growth by 50 %, were determined following a 72 h incubation period. None of the extracts exhibited substantial toxicity against hepatic cancer cells, as evidenced by IC50 values exceeding 0.5 mg/ml.

Regarding seed extracts, the IC50 value for LoVo and MCF-7 cells was specifically ascertained for ethanolic extracts of the seeds. Interestingly, *O. dillenii* seed extracts demonstrated cytotoxic effects even on hepatic cells, resulting in approximately 60 % cell survival at a con-centration of 0.5 mg/ml. Furthermore, ethyl acetate and water extracts from the seeds of Nador and Essaouira regions exhibited diminished cell viability for colon cancer cells, leading to around 30 % cell death at the same concentration. In the case of skin extracts, the IC50 value was determined for Diethyl ether extract against MCF-7 and LoVo cells. However, for HepG2 cells, treatment with the highest concentration resulted in a 60 % cell survival rate.

Conversely, ethyl acetate extraction displayed robust cytotoxic activity exclusively against breast cancer cells, inducing 40 % cell death at 0.5 mg/ml for the other tested cell lines. When applied at the highest concentration, the skin ethanolic extract exhibited only modest 20–30 % inhibition of cell growth. Remarkably, among the extracts obtained from the juice, notable cytotoxic activity was demonstrated by the IC50 value of the diethyl ether extract, exerting an im-pact across all analyzed cell lines (Katanić et al., 2019).

## 6 Simulation method

### 6.1 ADMET profile of the phytoconstituents

The ADMET (Absorption, Distribution, Metabolism, Excretion, and Toxicity) characteristics, which encompass absorption, distribution, metabolism, excretion, and toxicity, were determined for the six main compounds of *O. dillenii* using the QikProp module of the Schrödinger software (Rajagopal et al., 2020). Output PDB files were generated from this analysis, which can be examined and verified using suitable applications. This study revealed important physicochemical properties of the compounds, including their flexibility, molecular weight/size, hydrophobicity, bioavailability, permeability, and polar solubility. The assessment also included applying Lipinski's Rule of Five to evaluate the drug-likeness of the top compounds under investigation, indicating their potential for further development.

### 6.2 Preparation of the protein and ligands for molecular docking studies

The library of main compounds with antibacterial properties, sourced from *O. dillenii*, was obtained from the PubChem database (http://pubchem.ncbi.nlm.nih.gov) in the 3D coordinates of the Structure Data File Format (SDF). The three-dimensional structures of these compounds, along with the control ligand Gentamicin, were prepared for molecular docking studies. To achieve this, 3D and geometric optimizations and ligand energy minimization were carried out using controlled algorithms within Schrödinger 2021-2 (Elbouzidi et al., 2024, Loukili et al., 2023, Ouahabi et al., 2023). The LigPrep module, an integral part of Schrödinger's suite of tools, was utilized to add hydrogen atoms, eliminate salts, and perform ionization at a pH range of 7 ± 2. Subsequently, energy minimization was conducted using the OPLS4 force field.

**Fig. 5:**
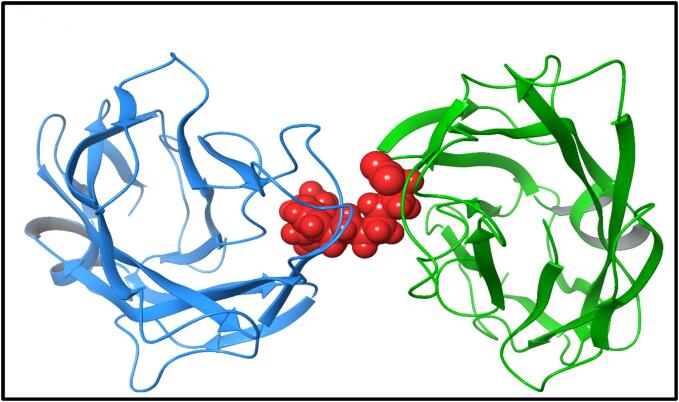
The 3D structure of the *Escherichia coli* complex and docked ligand in the binding site are shown in red. (For interpretation of the references to color in this figure legend, the reader is referred to the web version of this article.)

For molecular docking, the target organism *E. coli* was used, and the X-ray Crystal structure of the FimH lectin domain from *E. coli* K12 in complex with heptyl alpha-D-mannopyrannoside (PDBID: 4XO8) was chosen for docking purposes Fig. 5. *Escherichia coli* protein has been widely used as a standard in various antibacterial assays due to its extensive availability and the wealth of existing data. This protein structure had a resolution of 1.70 Å (Ludwig et al., 2021). The preparation of the protein structure, based on the Protein Preparation Wizard, involved the removal of ligand and water atoms, as well as the merging of non-polar hydrogens. The active site of *E. coli* was selected as the center for docking, and a grid box site was defined with coordinates x = 20 Å, y = 20 Å, and z = 20 Å.

The molecular docking calculations employed the extra precision (XP) glide score, which predicts binding free energy and ligand strain energy, aiding in the selection of docked poses. Energy minimization was performed with the OPLS4 force field, with a default RMSD limit of 0.3 Å (Fajriyah et al., 2023, Ludwig et al., 2021). Subsequently, the protein structure was minimized, and the conformation with the lowest binding energy was chosen as the result. To visualize the interaction of the ligand with active site residues, the selected conformation was converted into a two-dimensional diagram.

In summary, this process involved preparing ligands and protein structures, setting up the docking parameters, performing molecular docking calculations, and analyzing the interactions between the ligands and the active site of *E. coli* using computational tools and algorithms pro-vided by the Schrödinger software suite.

## 7 In silico ADMET predictions of pharmacokinetic studies

ADMET modeling has gained significant traction in pharmaceutical research for drug development due to its high throughput and cost-effectiveness. The results of ADMET predictions for the main compounds of *O. dillenii* are presented in Table 2, Table 3. The six compounds, linoleic acid, palmitic acid, α-tocopherols, betanin, kaempferol, and quercetin, given in these tables are the most abundant and biologically significant phenolic constituents of *Opuntia dillenii*. Lipinski's Rule of Five, a fundamental guideline for assessing the drug-likeness of compounds, was applied to evaluate key physicochemical properties of the compounds (Merzouki et al., 2023).

**Table 2:** In silico Lipinski's rule of five for the main compounds of *O. dillenii*.

Compound name	Molecular weight	Donor HB	Acceptor HB	QPlogPo/w	Rule of five
Linoleic acid	280.45	1	2	5.291	1
Palmitic acid	256.42	1	2	5.251	1
α-Tocopherols	430.71	1	1.5	8.970	1
Betanin	550.47	8	17	−0.793	3
Kaempferol	286.24	3	4. 5	1.041	0
Quercetin	302.24	4	5.25	0.367	0

**Table 3:** Predicted ADMET analysis of in silico main compounds of *O. dillenii*.

Compound name	SASA	FOSA	FISA	PISA	QPlogHERG	QPPCaco	QPlogBB	HOA
Linoleic acid	623.9	478.9	107.5	37.41	−2.551	239.64	−1.305	3
Palmitic acid	670.6	562.3	108.3	0.000	−3.293	235.48	−1.469	3
α-Tocopherols	910.9	848.0	35.72	27.18	−5.786	4539.6	−0.646	1
Betanin	839.5	225.1	516.2	98.11	−0.400	0.0020	−6.120	1
Kaempferol	507.4	0.000	241.1	266.6	−5.201	51.240	−1.893	3
Quercetin	519.2	0.000	288.5	230.7	−5.109	18.190	−2.419	2

Kaempferol and quercetin were found to strictly adhere to Lipinski's Rule of Five, with no violations in terms of molecular weight, hydrogen bond donors, hydrogen bond acceptors, and predicted octanol/water partition coefficient. However, some compounds such as linoleic acid, palmitic acid, α-tocopherols, and betanin violated specific criteria, such as QPlogPo/w > 5 or molecular weight thresholds. Further experimental investigation may be needed to better understand these violations' implications.

The main compounds of *O. dillenii* were analyzed using Schrödinger's QikProp module, which provides insights into various pharmacokinetic parameters. Parameters such as surface components (SASA, FOSA, FISA, and PISA), human-predicted qualitative oral absorption (HOA), QPPCaco (predicted apparent permeability of Caco-2 cells), and QPPCaco (gut barrier model) were within acceptable ranges, indicating excellent permeability and absorption characteristics. The predicted IC50 value for blockage of HERG K^+^ channels also met the specified requirements for most compounds, suggesting favorable pharmacological potential for them.

Overall, the main compounds of *O. dillenii* demonstrated good adherence to Lipinski's Rule of Five based on and predicted pharmacokinetic parameters revealing no major violations. This suggests these compounds possess drug-like characteristics and could serve as potential inhibitors. The findings of this study indicate that these in vitro compounds may be promising candidates for further preclinical evaluation in drug development.

## 8 Molecular docking analysis

A widely employed methodology in drug discovery to elucidate the intricate interactions between ligands and receptors is used in silico molecular docking techniques. Moreover, drug repurposing has emerged as a profoundly productive approach for identifying novel pharmaceutical entities, demonstrating the capability to curtail expenses and expedite the research trajectory (Diass et al., 2023). In the present investigation, we explored prospective compounds derived from *O. dillenii*, envisaging their potential to hinder the activity of *E. coli* through molecular docking analysis. The assessment of ligand-receptor affinities is quantified employing docking scores, wherein a more pronounced negative docking score connotes augmented binding affinity between the entities under scrutiny. Notably, the docking score constitutes a pivotal scoring function, instrumental in prognosticating the binding affinity of target-ligand interactions (as depicted in Table 4).

**Table 4:** Docking Scores of *Escherichia coli* (PDBID: 4XO8) with selected main compounds of Opuntia dillenii using XP docking.

Compound name	PubChem CID	Docking scores (kcal/mol)	Glide emodel (kcal/mol)
Betanin	57505033	−9.444	−62.609
Quercetin	5280343	−6.819	−56.867
Kaempferol	5280863	−5.790	−50.167
Linoleic acid	5280450	−5.706	−34.085
α-Tocopherols	319072904	−3.266	−41.891
Palmitic acid	985	−1.500	−30.816
Gentamicin	3467	−6.786	−40.364

The findings of our study unveil the elevated binding affinities of betanin and quercetin towards the active site of *E. coli*, surpassing the performance of the reference standard drug gentamicin, a noteworthy observation. The binding energies were computed as −9.444 kcal/mol for Betanin and −6.819 kcal/mol for quercetin, juxtaposed against gentamicin's binding energy of merely −6.786 kcal/mol. Kaempferol and linoleic acid, in a similar vein, exhibited commendable binding scores of −5.790 kcal/mol and −5.706 kcal/mol, respectively. In contrast, α-tocopherols and palmitic acid demonstrated diminished docking scores compared to the gentamicin control. Extensive analysis ensued, directed at comprehending the interaction modalities of the relatively steadfast chosen compounds (betanin and quercetin) within the confines of *E. coli's* active site, with a concerted effort aimed at unraveling the mechanisms of inhibition.

**Fig. 6:**
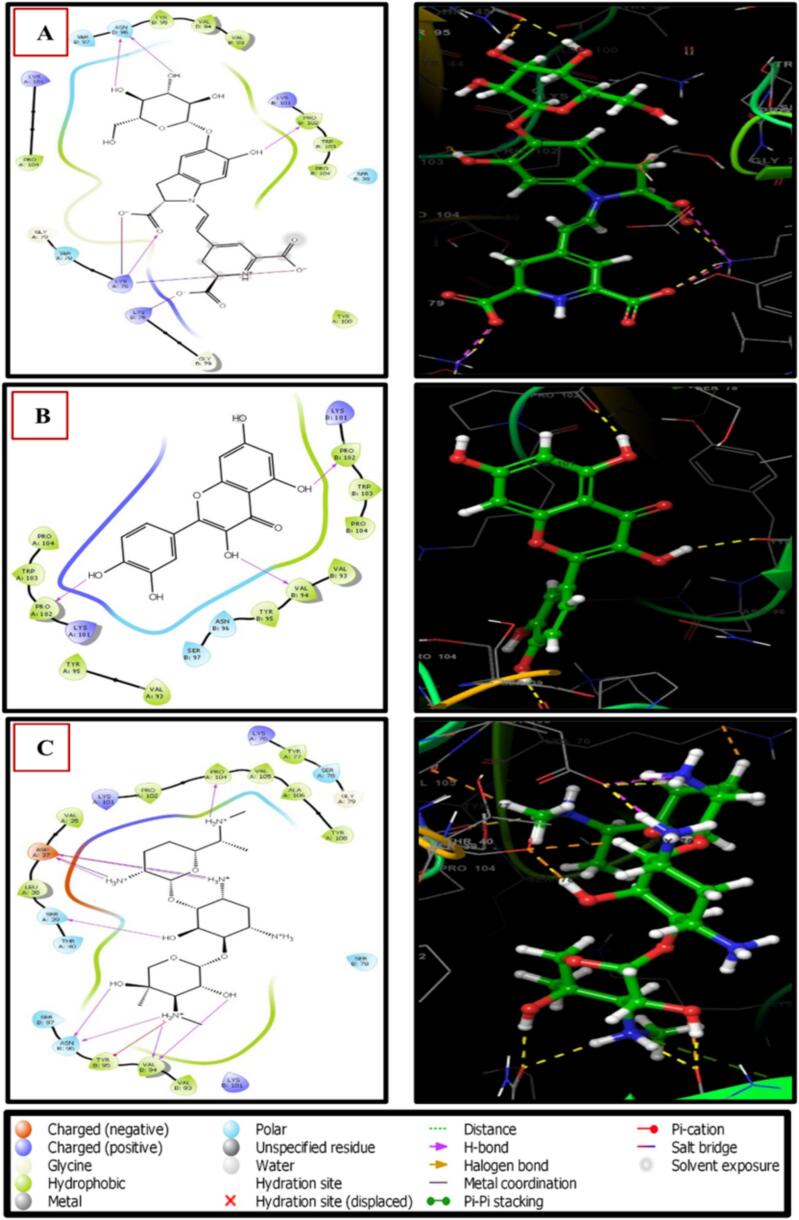
Two-dimensional interactions of the best results (A) Betanin, (B) Quercetin, and (C) control Gentamicin with the receptor of the *Escherichia coli* protein.

Fig.6 illustrates betanin, characterized by the most subdued binding energy and hence-forth the most plausible active inhibitor, manifested multifarious interactions with adjacent residues. It engaged in classical hydrogen bonding interactions with four amino acids, encompassing PRO102(B), LYS76(A), and 2ASN96(B), besides establishing three salt bridges with LYS76(B) and 2LYS76(A). Conversely, quercetin was stabilized by virtue of three hydrogen bonds with PRO102(B), PRO102(A), and VAL94(B), respectively. It is worth noting, however, that the avenue for further theoretical and empirical validations remains imperative to comprehensively ascertain and glean insights into the therapeutic effects of these compounds concerning antibacterial inhibition. Henceforth, the discourse underscores the compelling necessity for additional investigation and experimental validation to thoroughly explore the prospective antibacterial attributes of betanin and quercetin and unravel the nuances of their mechanisms of action.

## 9 Conclusions

This study has revealed that the examined *Opuntia dillenii* fruit extracts are notably rich in unsaturated fatty acids, with linoleic acid being the principal constituent, followed by palmitic acid. This composition makes these extracts suitable for various applications, including cosmetics and dietary lipid provision. The presence of these constituents within *O. dillenii* fruit extracts highlights their potential contribution to human health and well-being. Further analyses have elucidated the rich chemical composition of *O. dillenii* fruits, emphasizing the presence of bioactive molecules such as tocopherols and polyphenols. These compounds impart significant biological activity, bestowing the fruit with pharmacological, antioxidant, antidiabetic, antibacterial, and antifungal properties. The distinctive chemical variations observed across different *O. dillenii* extracts are influenced by factors such as environ-mental conditions, climatic and geographical variations, and species domestication.

The present investigation has effectively utilized the synergistic potential of molecular docking and ADMET predictions, resulting in a judicious and cost-effective strategy for identifying potential targets for antibacterial drug development. However, to definitively establish the therapeutic antibacterial potential of the primary compounds found in the prickly pear, future research must include comprehensive laboratory and clinical investigations. These efforts are crucial in confirming and expanding upon the potential medicinal attributes of these compounds, thereby enhancing our understanding of their antibacterial efficacy.

## CRediT authorship contribution statement

**Loukili EL Hassania:** Writing – review & editing, Writing – original draft, Supervision, Conceptualization. **Merzouki Mohammed:** Writing – original draft, Resources, Data curation. **Taibi Mohamed:** Writing – original draft, Resources, Investigation. **Amine Elbouzidi:** Writing – original draft, Software, Resources. **Belkheir Hammouti:** Writing – original draft, Formal analysis, Data curation. **Krishna Kumar Yadav:** Writing – review & editing, Writing – original draft, Supervision, Conceptualization. **Mohammad Khalid:** Formal analysis, Resources, Writing – review & editing. **Mohamed Addi:** Writing – original draft, Resources, Investigation. **Mohammed Ramdani:** Writing – review & editing, Writing – original draft, Data curation. **Pankaj Kumar:** Writing – review & editing, Writing – original draft, Resources, Data curation, Investigation, Software. **Jeong Ryeol Choi:** Formal analysis, Writing – review & editing.

## Declaration of Competing Interest

The authors declare that they have no known competing financial interests or personal relationships that could have appeared to influence the work reported in this paper.
